# Accuracy and quality of immunization data in Iran: findings from data quality self-assessment survey in 2017

**DOI:** 10.1186/s12913-019-4188-9

**Published:** 2019-06-11

**Authors:** Manoochehr Karami, Salman Khazaei, Abbas Babaei, Fatemeh Abdoli Yaghini, Mohammad Mehdi Gouya, Seyed Mohsen Zahraei

**Affiliations:** 1 Social Determinants of Health Research Center, Hamadan University of Medical Sciences, Hamadan, Iran; 2 Department of Epidemiology, School of Public Health, Hamadan University of Medical Sciences, Hamadan, Iran; 3 Department of Epidemiology & Biostatistics, School of Public Health, Tehran University of Medical Sciences, Tehran, Iran; 4 Research Center for Health Sciences, Hamadan University of Medical Sciences, Hamadan, Iran; 5 Center for Communicable Diseases Control, Ministry of Health and Medical Education, Tehran, Iran

**Keywords:** Immunization, Health surveys, Data quality self-assessment, Quality control, Data accuracy, Iran

## Abstract

**Background:**

The aim of this study was to assess the accuracy and quality of immunization data on the pentavalent (diphtheria, pertussis, tetanus, hepatitis B and *Haemophilus influenzae* type B (Hib)) and MMR vaccines as the administrative data of the expanded program on immunization (EPI) in Iran.

**Methods:**

We conducted a Data Quality Self-assessment (DQS) survey from October to December 2017. Standardized DQS tools were used to assess the accuracy of reported immunizations data and quality of the immunization monitoring system at the provincial level of the healthcare system including health houses, health posts, rural and urban health centers and district health centers. Multistage cluster random sampling with proportional to size (PPS) weights was used to select target provinces and related health units. Accuracy ratio, quality index (QI), completeness and relevant quality indices of first dose of MMR (MMR1) and third dose of pentavalent vaccines were reported. Corresponding period of the survey was limited to reported administrative immunization data during the first 6 months of 2016.

**Results:**

In relation to accuracy ratio, there was some evidence of under reporting of pentavalent (3rd dose) and MMR1 vaccines in health house units which were 100.94 and 101.1%, respectively. Completeness of reporting for both vaccines at different provincial levels was near 100%. However, the corresponding value for pentavalent (3rd dose) and MMR1 vaccines at the level of urban health centers was 96.67 and 94.17% respectively.

Among the five components of a monitoring system data usage and core output had the lowest QI scores in either rural or urban as well as district healthcare centers.

**Conclusions:**

Findings from our DQS survey reveals that administrative reporting of the immunization data was adequate at provincial and district levels of the healthcare centers. Although, addressing the existing concerns regarding timelines of the reporting by health authorities and staffs of EPI is warranted.

**Electronic supplementary material:**

The online version of this article (10.1186/s12913-019-4188-9) contains supplementary material, which is available to authorized users.

## Background

Immunization controls communicable diseases and is an essential component of public health policies. The vaccination program, as the most cost-effective health intervention eradicates the smallpox and other infectious diseases worldwide [1].

According to the expanded program of immunization (EPI) in Iran, children were vaccinated against ten diseases including bacillus calmette-guerin (BCG), oral poliovirus vaccine (OPV), hepatitis B (HBV), diphtheria, tetanus, pertussis (DTP), hepatitis B, diphtheria, tetanus, pertussis and *Haemophilus influenzae* type B (pentavalent) and measles-mumps-rubella (MMR) (Appendix 1). The vaccination schedule of EPI was attached in Appendix 1. Immunization services in Iran have been involved into the routine activities of the Primary Health Care (PHC) [2].

Checking the accuracy of data is important for the objectives of surveillance systems. The quality of reports depends upon the quality of primary data. Controlling data quality can also help improve the analysis of health report. The quality of surveillance data can potentially be affected by the degree of clarity of surveillance sheets, the quality of training and persons responsible for completion of the sheets [3]. Little studies about the accuracy of immunization and health system reports have been conducted in Iran despite the advancements in immunization programs. Vaccination program is prerequisite for monitoring vaccine effects, however just a few aspects were examined in this program [4, 5]. The qualitative aspects of immunization were disregarded. Despite the necessity of completeness and timeliness of the reports of immunization quality, these two major components are generally disregarded [6].

The World Health Organization (WHO) has recently introduced two major tools including DQA and DQS to evaluate the immunization data [7]. In 2003, the Global Alliance for Vaccines and Immunization (GAVI) released a tool called Data Quality Audit (DQA) to test the quality, accuracy, completeness and timeliness of immunization data. Ensuring the allocation of resources in line with the objectives of this union is another goal of this tool [5]. A study on 41 low-income countries showed that less than 80% of reports were accurate in half of those countries during 2002–2005 [6].

Data Quality Self-Assessment (DQS) is another tool published by WHO in 2005 [8]. The DQS is a flexible tool to evaluate the qualitative and quantitative aspects of the immunization monitoring system in different levels of healthcare system In fact, it helps countries identify weaknesses and strengths of the immunization program at different levels, and ultimately lead to improving the program and immunization services.

National Immunization Program has been one of the most successful health interventions in Iran [9]; however, 95% of vaccination coverage could not stop the scattered outbreak of measles in the country [10, 11]. With regard to the acceptable indices of immunization coverage in the Islamic Republic of Iran, ensuring the accuracy of the administrative reports on immunization at different levels of the healthcare system seems prudent. Therefore, the present study aimed to evaluate the accuracy of the immunization reports as well as the quality of the immunization monitoring system (for children under 1 year old) at different healthcare levels during the first 6 months of 2016. Given the convenience of access to demographic information on the denominator for children less than 1 year of age and considering the requirements for the calculation of immunization indices, we decided to investigate the accuracy and quality of immunization data on 3rd dose of pentavalent vaccine and 1st dose of MMR vaccines in Iran.

## Methods

The standard methodology of DQS was proposed by WHO [5]. This study assessed the immunization data include administrative reporting of the third dose of pentavalent (pentavalent3) vaccine (diphtheria, pertussis, tetanus, hepatitis B and *Haemophilus influenzae* type B (Hib)) (at the age of 6 months) and first dose of MMR (MMR1) vaccine (at the age of 12 months) among 900 children in the first 6 month (January–June) of 2016.

### Study setting

**Fig. 1:**
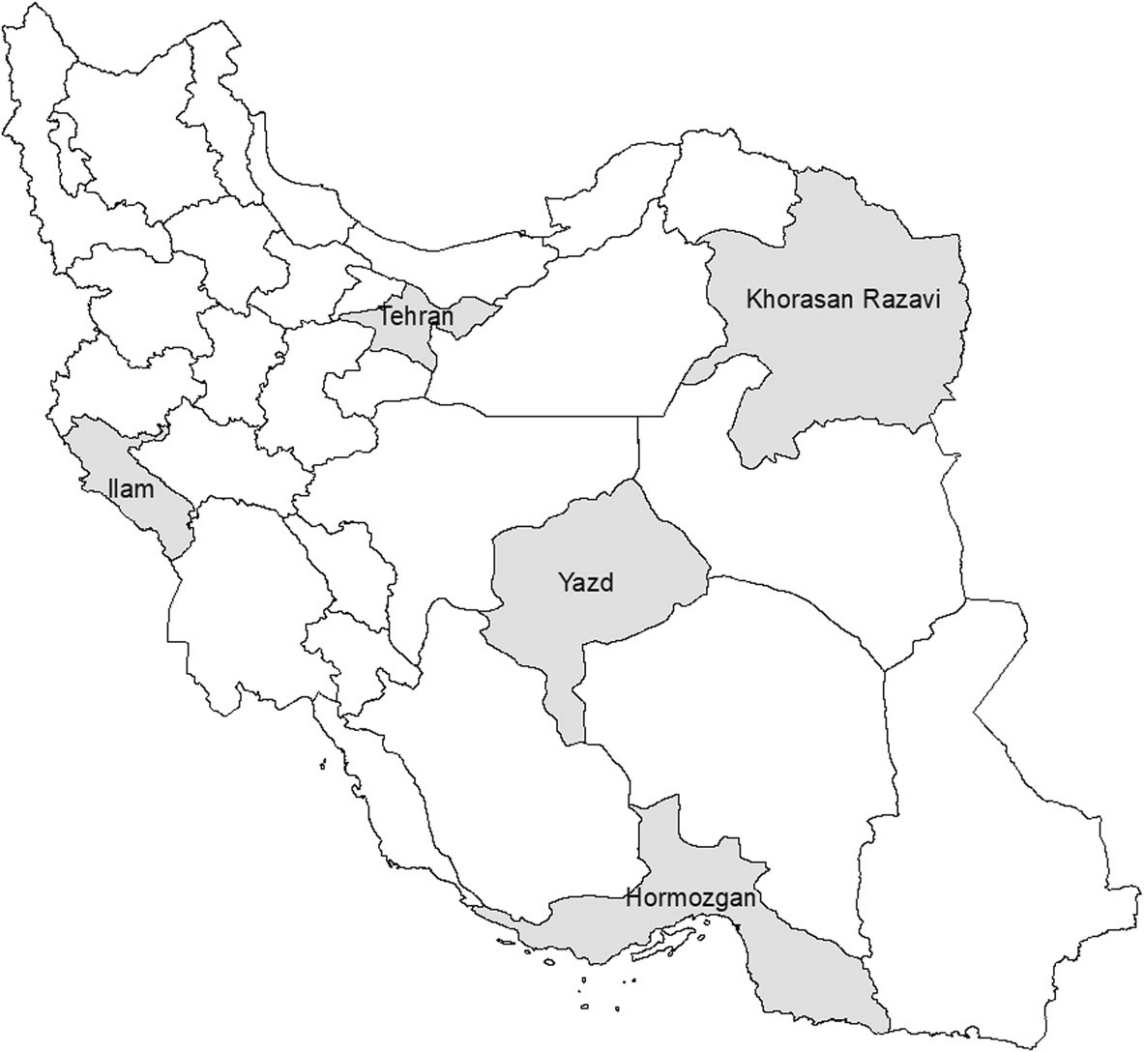
Geographical location of studied provinces in DQS survey. (Source: This map has been depicted by authors using Arc Vie Map software)

According to the sampling method, we investigated two district health centers throughout five provinces include Ilam, Khorasan Razavi, Hormozgan, Tehran and Yazd. The geographical location of the included provinces has been shown in Fig. 1. As well distribution of study populations according to 2016 census are presented in Appendix 2.

### WHO DQS tool

The DQS is a flexible toolbox of methods to evaluate different aspects of the immunization monitoring system at different health unit levels. By this toolbox data accuracy at different levels and some quality issues like availability of vaccination cards, use of tally sheets, directly-observed recording and reporting practices are assessed through a self-designed questionnaire (Additional files 1, 2, 3, 4 and 5). By identification of strengths and weaknesses, practical recommendations are made with the aim to improve the use of *accurate, timely* and *complete* data for action at all levels. Financial support from GAVI has contributed to the design and testing of the DQS [8].

### Sampling scheme for healthcare units (HUs)

We used multistage cluster random sampling with a proportional to size (PPS) weights for selecting HUs. A sequential procedure is described below:

1. Each of the medical universities proportional to the population of children under 1 year was weighted. So the university with higher frequency of children under 1 year had several times chance to choose. Ultimately five medical universities were selected randomly.
2. Two district health centers were selected randomly for each medical university (primary sampling unit)
3. Three rural health centers, two urban health centers (UHC) and one health post (HP) were selected randomly of the covered rural and urban health centers for each district health center (secondary sampling unit)
4. For each rural health center (RHC) one health house was selected randomly (Tertiary sampling unit)

**Fig. 2:**
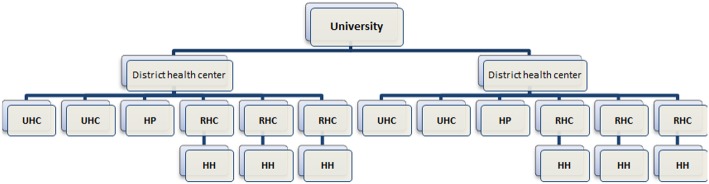
List of investigated health units covered by each university at provincial level. Abbreviations for Fig. 2: HH: Health house, UHC: urban health centers, RHC: rural health center, HP: health post

In total per all included medical universities in the study, 30 health house, 20 urban health centers, 10 health posts, 30 rural health centers and 10 district health centers were choose (Fig. 2).

### Sampling methods to assess the accuracy of recording

Registration folders for immunization of target groups in vaccine inoculation units (health houses, health posts and vaccination ward of urban health centers) were considered as the sampling frame. Names of 12 to 23 months old children were assigned one number from one forward. In continue through systematic random sampling 15 children were selected from each health unit.

### Outcome measures definition

The standard formulas for calculation of accuracy ratio, completeness and timeliness were adapted from DQS methods as follow.

Accuracy ratio = Number of vaccinations verified or re-counted from a source at one level/ compared to the number of vaccinations reported by that level to more central levels. Completeness = (Number of reports received / number of reports expected during a period of time) × 100. Timeliness = (Number of reports that were received on time (by the deadline set by the EPI office) / number of reports expected during a period of time) × 100.

### Accuracy of reported immunizations data

For assessment of the accuracy of reporting, according to the guidelines [8], the date of vaccination must be recorded on both children’s vaccination card and the immunization logbook provided to health house, health post, and vaccination ward of urban health centers. Moreover, the administered dose should be recorded on an appropriate sheet allowing for the easy re-counting of all doses provided. These Health units’ monthly reports inculcated vaccines to the upper level (rural or urban health center). In order to the assessment of the accuracy of reporting accuracy in this level, the number of administered doses from the tally sheets for each month was compared with the monthly report to the higher level. For rural/urban and district health centers cumulative monthly reports of lower levels were compiled to the monthly report to higher levels.

For assessment of the accuracy of reporting in the community, following selection of 15 children (the required sample size), the interviewers paid them a home visit and the dates of MMR1 and Pentavalent3 vaccines inoculation were recorded based on the vaccine card.

Immunization records in the healthcare unit were then checked to detect any discrepancy between the information registered in the child’s vaccination card and the immunization logbook. Finally accuracy ratio was calculated based on the number of vaccinations verified or re-counted from a source at one level to the number of vaccinations reported by that level to more central levels.

Completeness of HU reporting was calculated for the first 6 months (January–June) of 2016 based on the numbers of HU reports available at the upper level for a given period were re-counted. This is referred to as an indicator of the availability of reports, defined as the proportion of reports physically available at the time of the assessment for a this time period divided by the total number of reports expected to be available.

The timeliness of HU reporting, the dates of sending reports to the upper level were looked. According to the national policy, this date can be + 3 days for health houses, + 5 days for rural and urban health centers and + 12 days for district health centers. Therefore, by calculating the reports that were sent on the defined time interval, the timeline rate of reports were assessed at each level.

### Quality of the immunization monitoring system

For assessment of the quality of the immunization monitoring system at different levels, the standardized WHO questionnaire was used. We have used hard copy questionnaires and asked trained assessors to complete the questionnaires. These questions/observations/tasks were grouped into six components of the monitoring system including: Recording, Archiving, Reporting, Demographic information, Core output / analyses and Evidence of using data for action. Questions were modified for each healthcare unit level. For each question, a score was considered. A “no” scores 0, a “yes” scores from 1 to 3 according to its importance, and an “NA” was not recorded in the denominator.

Scores were calculated for each of the identified components, with the number of points corresponding to correct answers as the numerator and the number of possible scores as the denominator. The quality index (QI) was calculated by the following formula:

QI = scores for all questions answered “yes” / sum of maximum scores that could be obtained.

Frequency tables and chart were used for presenting the data. Data were analyzed using Microsoft *Excel* 2010 software.

## Results

### Findings on accuracy ratio

**Fig. 3:**
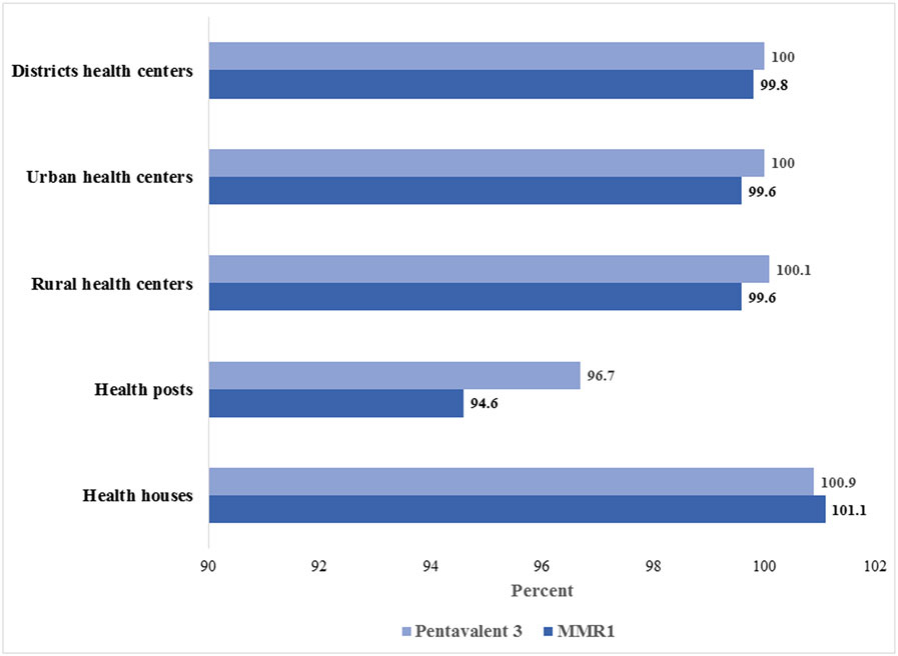
Proportion of re-counted third dose of pentavalent and first dose of MMR immunizations which reported by administrative health levels to their higher level in first 6 months of 2016

As shown in Fig. 3, there was some evidence of under reporting for pentavalent (3rd dose) in health house units (100.94%) and rural health center units (100.1%); while over- reporting was observed in health post units (96.7%). The accuracy ratio for both urban and district health centers was 100%.

Apart from health house units with under- reporting, there was some degree of over- reporting for MMR1such a way that the accuracy ratio for rural health centers, urban health centers, health posts and district health centers were 99.6, 99.64, 94.68 and 99.87%, respectively.

### Findings on accuracy in the community

A total of 450 children were assessed for accuracy ratio of Pentavalent3 and MMR1. Comparison of child’s vaccination card with immunization registry for pentavalent (3rd dose) and MMR1 revealed an accuracy ratio of 96.55 and 94.35% for health house units; and 93.62 and 92.11% for urban centers, respectively.

### Completeness and timeliness of the reporting

As shown in Table 1, completeness of the reporting of the 3rd dose of pentavalent were 100% for health post, rural health center and district health center units, while it was 99.45% for health houses and 96.67% for urban health centers. On the other hand, completeness of MMR1reporting was 98.89% for rural health centers, 94.17% for urban health centers, and 100%for other health units. The lowest percentage of timely reporting belonged to district health centers (83.33%), urban health centers (84.17%), and rural health centers (85%) for pentavalent (3rd dose); and to district health center (83.33%) for MMR1.

**Table 1:** Completeness and timeliness of pentavalent (3rd dose) and MMR1 vaccine reporting in different administrative health units

Health unit	Completeness of reporting (%)		Timeliness of reporting (%)	
	pentavalent (3rd dose)	MMR1	pentavalent (3rd dose)	MMR1
Health house	99.4	100	94.4	94.4
Health post	100	100	93.3	93.3
Rural health center	100	98.8	85	85.5
Urban health center	96.6	94.1	84.1	84.1
District health center	100	100	83.3	83.3

Pentavalent: diphtheria, pertussis, tetanus, hepatitis B and *Haemophilus influenzae* type B (Hib); MMR1: Measles-Mumps-Rubella

### Findings on quality of the monitoring system

**Fig. 4:**
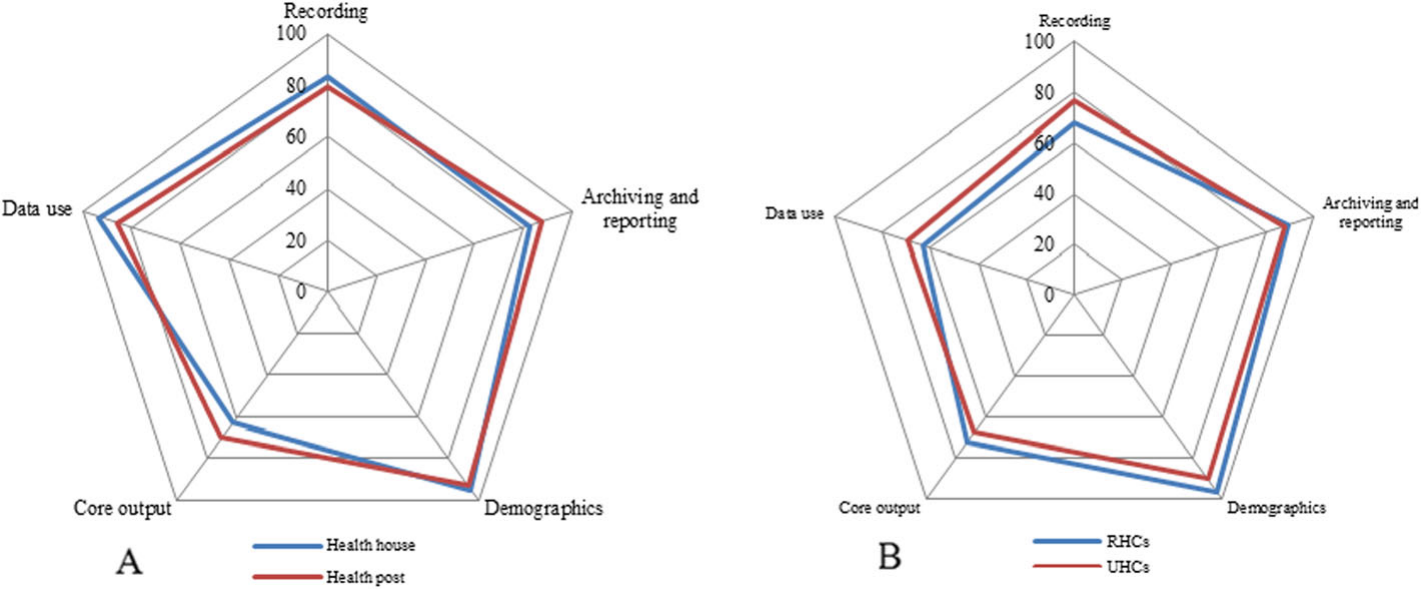
Quality indices for five components of a monitoring system at health house/posts (**a**) and health centers (**b**) level

As shown in Table 2 & Fig. 4a, Quality index scores varied in different components of the monitoring system. QI was ranged from 62.7% for core output to 94.7% for demographics at health house levels and ranged from 70% for core output to 92.9% for demographics at health posts levels.

**Table 2:** Quality indices (QI) for five components of a monitoring system at house/posts level

Health unit		Recording	Archiving and reporting	Demographics	Core output	Data use
Health house level	Maximum score	450	450	438	432	480
	Acquired score	376	370.5	415	271	447
	QI	83.6	82.3	94.7	62.7	93.1
Health post level	Maximum score	150	150	141	150	180
	Acquired score	119	131	131	105	180
	QI	79.3	87.3	92.9	70	86.1
Total	Maximum score	600	600	579	582	660
	Acquired score	495	501.5	546	376	602
	QI	82.5	83.6	94.3	64.6	91.2

Qualities of the monitoring system at rural/urban health centers as the second level of vaccination service are presented in Table 3 & Fig. 4b. For RHCs level, QI was ranged from 63.1% for data use to 96.3% for demographics. In this level QI for recording and core output was 68.3 and 72.5% which was not suitable. QI for UHCs level was ranged from 63.4% for data use to 90% for demographics. Totally in this level of vaccination service, core output and recording components with the QI of 65.6, 70.6 and 71.5% had the lowest score, respectively.

**Table 3:** Quality indices (QI) for five components of a monitoring system at health centers level

Health unit		Recording	Archiving and reporting	Demographics	Core output	Data use
RHCs level	Maximum score	246	795	270	360	360
	Acquired score	168	709	260	261	227
	QI	68.3	89.2	96.3	72.5	63.1
UHCs level	Maximum score	150	525	180	231	420
	Acquired score	115	462	162	156	166.5
	QI	76.7	88	90	67.5	63.4
Total	Maximum score	396	1320	450	591	600
	Acquired score	283	1171	420	417	393.5
	QI	71.5	88.7	93.8	70.6	65.6

Quality indices at district health centers level for recording, archiving and reporting, demographics, core output and data use were 88.33, 95.24, 94.16, 77 and 85%, respectively.

## Discussion

Improving the quality of the reported data in immunization program is essential [12]. Thus, WHO has recently introduced DQS tool to examine the immunization coverage for countries. In this study, DQS method was used to evaluate the accuracy of immunization reports as well as the quality of the immunization monitoring system for children less than 1 year old at different healthcare levels in Iran.

Based on the results, quality index scored from 62.7 to 94.7% for core output and demographics at health house levels, respectively. The corresponding values ranged from 70 to 92.9% for demographics at health posts levels. Moreover, there is a negligible proportion of under/ over reporting for pentvalent 3 and MMR1 in different administrative healthcare levels. The accuracy ratios of the two vaccines were about 95 and 93% for health house and health post levels, respectively. The percentage of completeness for vaccine reports were 100% (at all levels), 95% (urban health level) Also, the percentage of timeliness for reporting the administrative health units (health houses and health units) and other levels were nearly 95 and 85%, respectively. The lowest score of quality index belonged to core output in health house and health post units. The lowest QI scores belonged to rural and urban health centers as well as district health centers.

Although the results confirmed the adequacy of accurate information obtained from national immunization registration system, this survey identified major challenges in the national immunization monitoring system in terms of timelines of reporting and quality for core output and data use.

The results of studies aiming at measuring the accuracy and quality of immunization information systems on 41 low-income countries (30 African, 10 Asian and one Caribbean) showed that almost half of the countries obtained a low verification factor (VF) (80%) and nine countries had high VF and quality scores (QS). The most common weaknesses in the information systems of these countries were inconsistency of denominators used to estimate coverage, unavailability of guidelines, incorrect estimations of vaccine wastage and lack of feedback on immunization performance [6, 13]. *Adamki* et al. conducted a study in eight reproductive and child health units and health centers in Ghana to determine the accuracy and completeness of reported number of vaccinations. They found that the quality of data was generally poor [14]. In another study in Tunisia, the regional verification factor and quality index were estimated to be 85 and 55%, respectively [15]. According to the national data (DQS study) in Uganda in 2013, the quality of administrative vaccination data was not optimal, especially at the subnational level. Inaccurate data on administered doses of vaccine result from lack of knowledge of healthcare staff, standard tools for recording and reporting, and inadequate implementation of recommended practices for data collection, analysis, and use [16]. Inappropriate storage of reports and documents related to the immunization and subsequently data loss was the cause inaccuracy of Cameroon DQA [15].

Monitoring vaccination coverage is one of the most important components of any immunization program [17]. In the present study, there was a small percentage of under/over reporting, which could be affected by reported vaccination coverage by administrative health levels. The inconsistencies of data reporting is not a new issue [18, 19]. Lack of adequate supervision, feedbacks from higher levels, and inadequate incentives of healthcare staff were the main reasons of errors [18, 19].

Pressure from higher levels, inclusion of vaccination conducted outside of target group, reporting doses instead of immunizations, using non- standard tools to report daily vaccinations, miscalculation and loss of verifiable information are attributable to accuracy ratios less than 100%, and in addition to these factors, incomplete reports at the time of forwarding were considered as possible reasons for accuracy ratio higher than 100% [8]. Mostly, the concern relies on over reporting as it could potentially lead to under-vaccination. Thus, using smart software to record vaccination data, supervising the vaccinators’ performance, additional training can improve reporting quality. However, identifying the defects of field and providing the precise, efficient and useful immunization monitoring system is highly recommended.

Quality of the monitoring system was the other aspects of the immunization monitoring system assessed in the present study. The results of this study report the robust capacities and demographics; however, the system did poor in the areas of core outputs/analysis and data use. In fact, raising the capacity of the healthcare workers to manage, analyze, and use the vaccination data is the central component of DQS tool.

Insufficient motivation and inadequate training of healthcare staffs, inappropriate prioritization of activities due to integration of services; inadequate supervision by higher levels and limited financial resources can be considered as the common challenges of the low scores of core output and data use throughout all administrative healthcare levels [16]. The integration of multiple programs in the health system of the country and the change of priorities can be considered as the most important challenges in the quality of immunization quality in the present study.

It should be kept in mind that data quality and data use have a mutual connection, so as they can help drive, demand and use, and consequently motivate to create high quality data. If data are not used to monitor performance changes in target population, or damage stricken areas could not be identified [20].

In this study, we used the DQS tool for comprehensive assessment of immunization data quality in Iran. The output of this tool allowed the assessors to prove the system’s effectiveness by validating the data collected in different administrative healthcare levels.

Financial problems, sample size and a limited amount of archived data were the limitations of this study. Extending the duration of study and examining two different vaccines (pentvalent3 and MMR1) for each child could solve some limitations.

Despite some quantitative and qualitative weaknesses and strengths of the national immunization system, further study should be conducted to determine factors affecting poor quality in core output and data use. Doing DQS survey in other provinces with large samples can lead to more conclusive results.

## Conclusions

The output of DQS tool showed that information relating to accuracy ratio from national immunization registration system was adequate; however, there were some weaknesses in timelines of reporting and quality of core output and data use. Integration of DQS concept into the national training schedule, using appropriate software to record vaccination data, supervising the vaccinators’ performance, staff training can improve monitoring practices and management of immunization.

### Additional files

Additional file 1: Accuracy ratio of pentvalent 3 & MMR1 register in the community (PDF 52 kb)

Additional file 2: Accuracy ratio of pentvalent 3 and MMR1vaccines (PDF 9 kb)

Additional file 3: Standard questions to assess the quality of the monitoring system (PDF 139 kb)

Additional file 4: Standard questions to assess the quality of the monitoring system (PDF 200 kb)

Additional file 5: Timeliness and completeness of Pentavalent 3 and MMR1vaccines reporting (PDF 12 kb)

## Data Availability

The dataset of the current study is available from the corresponding author at a reasonable request.

## Appendix

**Table 4:** The vaccination schedule of EPI in Iran

Age	Vaccine
At birth	BCG, HBV, OPV
2nd month	OPV, pentavalent
4th month	OPV, pentavalent
6th month	OPV, pentavalent
One year old	MMR
18th month	MMR, DTP, OPV
6 years old	DTP, OPV

*BCG* Bacillus Calmette-Guerin, *HBV* Hepatitis B, *OPV* Oral Poliovirus Vaccine, *DTP* Diphtheria, Tetanus, Pertussis, Pentavalent: Hepatitis B, Diphtheria, Tetanus, Pertussis and *Haemophilus influenzae* type b, and *MMR* Measles-Mumps-Rubella

**Table 5:** Distribution of study populations (According to 2016 census)

Province	Population (2016 census)					under one year of age population (2016 census)		
	Male	Female	Urban	Rural	Total	Urban	Rural	Total
Ilam	295,199	284,959	395,263	184,444	580,158	6570	3110	9689
Tehran	667,362	6,593,965	12,452,230	814,698	13,267,637	177,577	6970	191,171
Yazd	586,013	552,520	971,355	166,724	1,138,533	20,511	3189	23,700
Khoasan Razavi	3,245,185	3189,316	4,700,924	1,73,121	6,434,501	93,270	40,702	133,978
Hormozgan	906,814	869,601	971,822	802,512	1,776,415	22,110	19,733	41,917

## Abbreviations

DQA: Data Quality Audit
DQS: Data Quality Self-assessment
GAVI: Global Alliance for Vaccines and Immunization
HU: Health unit
PPS: Proportional to size
QI: Quality index
VF: Verification factor
WHO: World Health Organization
