# Supplementary material for: Accuracy and quality of immunization data in Iran: findings from data quality self-assessment survey in 2017
Source: BMC Health Serv Res. 2019 Jun 11;19:371. doi: 10.1186/s12913-019-4188-9 (PMC6560874; doi:10.1186/s12913-019-4188-9)
Supplement: Supplementary file 1 — Accuracy ratio of pentvalent 3 & MMR1 register in the community (PDF 52 kb) [file 12913_2019_4188_MOESM1_ESM.pdf]

Accuracy ratio of pentvalent 3 & MMR1 register in the community

Row	Register No.	Name of child	Date of birth			Date Pentavalent 3 vaccination (register)	Date MMR1 vaccination (register)	Card possession		Date Pentavalent 3 vaccination	Date MMR1 vaccination	Pentavalent 3 vaccination history		MMR1vaccination history		Verified Pentavalent 3		Verified MMR1	
			D	M	Y			Yes	No			Yes	No	Yes	No	Yes	No	Yes	No
1																			
2																			
3																			
4																			
5																			
6																			
7																			
8																			
9																			
10																			
11																			
12																			
13																			
14																			
15																			

Name of the interviewers______________________ Team______________________________ Date of interview ____
