# Supplementary material for: Accuracy and quality of immunization data in Iran: findings from data quality self-assessment survey in 2017
Source: BMC Health Serv Res. 2019 Jun 11;19:371. doi: 10.1186/s12913-019-4188-9 (PMC6560874; doi:10.1186/s12913-019-4188-9)
Supplement: Supplementary file 2 — Accuracy ratio of pentvalent 3 and MMR1vaccines (PDF 9 kb) [file 12913_2019_4188_MOESM2_ESM.pdf]

Accuracy ratio of pentvalent 3 and MMR1vaccines

Year	Month	Pentvalent 3		MMR1	
		**Recounted**	**Reported**	**Recounted**	**Reported**
2015	January				
	February				
	March				
2016	April				
	May				
	June				
Sum					
