# Supplementary material for: Accuracy and quality of immunization data in Iran: findings from data quality self-assessment survey in 2017
Source: BMC Health Serv Res. 2019 Jun 11;19:371. doi: 10.1186/s12913-019-4188-9 (PMC6560874; doi:10.1186/s12913-019-4188-9)
Supplement: Supplementary file 3 — Standard questions to assess the quality of the monitoring system (PDF 139 kb) [file 12913_2019_4188_MOESM3_ESM.pdf]

Standard questions to assess the quality of the monitoring system

B. Health units' level

	Question	This question should be designed to find out:	Weight (from 1 to 3)
**Recording component (HU)**			
1	Are there tally sheets for infant vaccinations on the desk (or easily available) and do they have entries for the last immunization day?	The main concern is evidence of use of availability (official form) and tally sheet.	
2	Are registers used for recording individual information about child immunizations?	Each HU should have a book or register where each immunization history can be registered and traced back.	
3	Can a child's vaccination history be easily and rapidly retrieved in the registers?	A new dose should not be entered as a complete new entry but entered in the location where previous doses have been entered. Score 0 if the register is used as a new entry for any immunization.	
4	Are registers (or pre-printed forms) used for recording individual information about women's TT immunizations?	There may be registers or health cards if cards kept in HU.	
5	Observe at least five vaccinations:<br><br>Were all vaccinations well registered on the child health card/tally sheet/register?		
6	Are individual immunization records used, updated and given to the child's caretaker at the time of the immunization visit?	Blank cards should be available in the HU. Immunization cards are often integrated in "Road to Health" or other health cards.	
7	Are vaccine receipts recorded in a vaccine ledger?	Check against available stock (count doses in the refrigerator).	
8	Ask the child's caretaker: Do you know the expected date of receiving vaccine?	Find out whether the expected dates are known.	
9	Is the ledger up to date for all vaccines and/or a selected vaccine?	Up to date = all receipts and issues recorded immediately.<br><br>Check against stock (in therefrigerator). Compare the date of last entry and the date of the last immunization session.	
10	Is the receipt of a selected vaccine in the ledger complete for the entire year?		
11	Is there a log (vaccine ledger/stock card) for receipt/issuing of syringes supplied (AD/non-AD reconstitution syringes)?	Can perform a stock check.	
12	Does the HU record vaccine batch-number and expiry date?		
13	Are all individual recording forms available for the entire previous year?	Individual recording form = tally sheet or register.	

14	Did every person doing the child immunization card exercise get a perfect score for: DTP1<br>DTP3<br>measles?	Need to define how the scoring will be if a perfect score is not obtained.	
15	Is the cold chain temperature monitoring chart completed daily?	Check the chart and compare the latest reported temperature with the actual temperature in the refrigerator.	
**Reporting component (HU)**			
16	Have all reports for the previous year been signed by the officer-in-charge or officer authorized to submit the HU report?	Score for example:<br>If >50% reports are signed score "Yes".<br>If <100% discuss with the HU why some have not been signed.	
17	Does each report from the previous year have at least one date stamped or written on it by the HU – either as "signed date" or "compiled date"?	If >50% reports are signed score "Yes".<br>If <100% discuss with the HU why some have no data stamped or written in. This can be answered at district level.	
18	Are the HU reports correctly filled in?	Select a number of fields to be checked in all HU reports and check whether these have been filled in correctly.	
19	Are health staff aware of standard operating procedures and the necessary forms to complete if there is a report of a severe AEFI?	Ask health staff what is supposed to be done if a child becomes severely ill or dies after a vaccination. Ask to see any forms that are to be used.	
20	Are the HU reports completely filled in?	Select a number of fields to be checked in all HU reports from the previous year and check whether these have been filled in.	
**Archiving component (HU)**			
21	Can copies of all previous reports from this HU be found in the HU?	For current and previous year.	
22	Is there one location where the previous immunization reports and recording forms are stored?		
23	Are the reports of the HU organized in a file by date?	The main concern is that the reports are easily retrievable.	
24	Are HU reports available for the entire year?		
25	Are the child registers available for all periods of the previous year?		
26	Can all tally sheets covering the previous year be found?		
27	Are registers for TT vaccinations to pregnant women available for the entire previous year?		
28	Is the latest feedback on data from district easily available?		
**Demographic information component (HU)**			
29	Does the HU have data on the number of infants born in its catchment area?	The number of births should be different from the previous year. Discuss if there is a difference with the denominator available at more central level. Discuss ways to collect denominator information from community	

		(e.g. birth register), data from national immunization days (NIDs), or other sources. Discuss if the target was set up by the district or HU level.	
30	Does the HU have a target set on the number of children that should be vaccinated during the calendar year?	Discuss how realistic the value is.	
31	Does the HU have a system that allows the collection of information on new births in the community?	This may include community health workers, traditional birth attendants, outreach clinics, etc. A system means (a) organized way to collect the information in every village/community and (b) a written track available at the HU.	
32	Does the HU have a target by type of strategy (fixed/outreach/mobile) with a map showing the catchment area by strategy including the outreach villages?		
**Core outputs/analysis (HU)**			
33	Does the HU have a (target) number of children that it strives to vaccinate during a calendar year or a reporting period?		
34	Is there a mechanism in place to track defaulters?	Can be an appropriate use of a correctly filled register, tickler file, etc. When was the last time a child was followed up?	
35	Does the HU have achievements split by type of strategy – fixed/outreach/mobile?	It is important is to know the proportion of numbers actually reached by each strategy.	
36	Does the HU have an up-to-date chart or table (preferably on display) showing the number of vaccinations by report period for the current year?	Monitoring coverage chart – must be UP TO DATE.	
37	Is there a monthly chart/graph of VPD cases (broken down by VPD)?	How do these data correspond to coverage data (i.e. more cases in areas with poor coverage). When was the last VPD outbreak? Was it investigated? Why did it occur?	
38	Does the HU monitor drop-out rate?	Preferably on display with the same monitoring chart as the coverage one, but score 1 if the health worker can tell you the drop-out rate for his HU. Discuss the importance and reasons for drop-outs.	
39	Does the HU monitor vaccine wastage?	Discuss the reasons for wastage and any ways it might be reduced. Discuss whether the health worker knows how much the vaccine wastage is and how it can be calculated.	
**Evidence of using data for action component (HU)**			
40	Is there a mechanism in place to track defaulters or vaccine doses that are due?	Check how the HU can know when a child should return for a vaccine dose (e.g. DTP,	

		Hib, HepB, polio, measles or yellow fever vaccine dose).	
41	Is there a map showing the catchment area, including the outreach villages?	This ideally shows strategy type.	
42	Are areas of low access identified and is there evidence of actions taken to deal with this?	If there is low access (evidenced by low BCG or DTP1 coverage), how does it relate to the effectiveness of the three strategies (fixed site, outreach and/or mobile teams).	
43	Have reasons for any high drop-out been identified; are there plans/actions to deal with this?	Are there any managerial practices that can be changed?	
44	Have actions been taken on the last feedback from the district?		
45	Is there interaction with the community regarding immunization? Ask for information on "what" and "when"?	Are health staff actively involved in any community committees or meetings on health, investigations of outbreaks or any rumours of AEFIs, etc?	
