# Supplementary material for: Accuracy and quality of immunization data in Iran: findings from data quality self-assessment survey in 2017
Source: BMC Health Serv Res. 2019 Jun 11;19:371. doi: 10.1186/s12913-019-4188-9 (PMC6560874; doi:10.1186/s12913-019-4188-9)
Supplement: Supplementary file 4 — Standard questions to assess the quality of the monitoring system (PDF 200 kb) [file 12913_2019_4188_MOESM4_ESM.pdf]

Standard questions to assess the quality of the monitoring system

A. Monitoring level

	Question	Comment	Weight (from 1 to 3)
**Recording component**			
1	Are vaccine receipts and issues recorded in a vaccine ledger?	To assess appropriate record-keeping of vaccine receipts and issues.	
2	Does the district manager know the annual vaccine requirements for the district?		
3	Is the lot number/batch number and expiry date of the vaccine recorded?		
4	Is the current ledger up to date for a given vaccine (or vitamin A)?		
5	Is there a log of syringe supply and delivery to HU up to date?	Is the stock available identical to the quantity recorded in the register (count).	
6	Are district staffs aware of standard operating procedures to record a severe adverse event following immunization (AEFI)?		
7	Do the district's reports (found at district level) have at least one date stamped or written on them?	Determine what proportion of correct HU reports you would need to answer "Yes".<br>Define for which period (e.g. for the previous year)<br>Define the date significance:<br>the date the report was signed<br>the date of receipt at district level (stamped or written on it by the district office)	
8	Are the district reports (that are sent to more central levels) completely and correctly filled in?	To select a number of fields to be checked in all district reports and check whether these have been correctly filled in.	
**Archiving component**			
9	Is there a separate file or sub-file for each HU and are the reports inside filed by date?	Storage should facilitate retrieval and monitoring (and be well organized).	
10	Have all HU data from the previous month been processed?		
11	Are supervisory reports available?		
12	Are copies of the last feedback to the health facilities easily available?		
13	Can copies of all district reports (that were sent to more central levels) be found?		
**Computerized archiving**			
14	If the district is computerized is the last date of backup within one week? (look at the date the file was created on the diskette)	Check diskette for last saved date; look at the file creation date.	

15	Can the official immunization tabulations for the previous year be reproduced from an archived electronic file?	To check official immunization tabulations = final summary of previous year data.	
16	If more than one computer has immunization data, is there either a functioning network or a written, well-organized method of data transfer? (If yes, read it.)		
17	Is the date of printing /production on every tabulation/chart produced or, if the data is archived, is there a date showing when the archived file was created?		
**Reporting component**			
18	Have the district reports of the last year month been sent on time?		
19	Is the procedure for dealing with late reports known and applied?		
20	Did all the monthly (quarterly) reports from the HUs use the same form/format for the current year?		
21	Is there a system for investigation of individual reports of adverse events following immunization (AEFI) from the district to the higher level functioning/operational?	Serious AEFIs should be rapidly reported and investigated.<br>Investigators should be looking for any evidence of programmatic error that must be rapidly corrected and/or rumours that cause problems.	
22	Did all the visited HUs report adequate supply of administrative forms tally sheets/reporting forms/health cards?		
**Demographic information component**			
23	Is the district denominator for immunization of infants and pregnant women (and school children, if applicable) known?	Known: the interviewed senior staff member should be able to tell (without looking) approximately how many infants the district contained and how the figure was calculated (if relevant).	
24	Is there a district map of the catchment area showing HUs and providing immunization strategies (fixed, outreach, mobile)?	Ideally, the map should include denominator, target, type of strategy.	
25	Is the proportion of infants per strategy-type known for the district?	Usually fixed – outreach – mobile team, etc. This should be used in a district microplan.	
26	Has the same denominator for child immunization been used on different tabulations, reports, charts, tables, etc?	Indicate for which year.	
27	Are the denominators used in the current year different from the denominators used in the previous year?	Should be different from previous year.	
28	For the previous year, is the district denominator value (for child immunizations) found at the district the same as that used at national level?		
29	Is the denominator established independently?	The denominator should be established	

		independently from locally set-up targets.	
30	Are the denominators of each HU available for the previous year?	Answer “Yes” if available. Totals should add up to the district total.	
31	For the previous year, has only one denominator value (check at least total population) been seen in all health projects/programmes?	Check with various initiatives (e.g. polio, nutrition, malaria) whether the denominator is consistent at district level.	
**Core outputs/analyses component**			
32	Is there a target number of children that the district strives to vaccinate during a calendar year or reporting period?		
33	Is there a chart or table of immunization coverage by report period for the current year (monitoring chart)?	Is it on display?<br>Is it UP TO DATE?<br>Does it cover all antigens?	
34	Is the completeness of the immunization reporting from HU recorded and monitored at district level?	District staff should be able to describe what percentage of HU reports was received on time, received but not on time, and not received at all during the previous year or the last months.	
35	Does the district record and monitor timeliness for HU immunization reporting?	District staff should be able to say (based on printed information) what percentage of HU reports was received on time during the previous year or the last months.	
36	Is the drop-out rate monitored?	Discuss the importance and reasons for drop-outs. Are there managerial practices that could be changed to reduce the drop-out rate?	
37	Is there monitoring of HU/district vaccine wastage?	Discuss the importance and reasons for wastage.	
38	Is there a graph by month of the incidence of vaccine-preventable diseases (VPDs) –<br>broken down by VPD?	How do these data correspond to coverage data (i.e. more cases in areas with poor coverage). When was the last VPD outbreak? Was it investigated?<br>Why did it occur?	
39	Is an up-to-date chart/table of the completeness of the current year’s immunization data available?	Completeness = reports received or not received from the HUs. (Here the score 0/1 is only for completeness.)	
40	Is the HU performance monitored at the district level?	Monitoring of HUs: graph/figures showing how all HUs are performing during the current year.	
41	Are supervision activities monitored?	A written schedule of supervision that includes visiting every HU within a specific period of time.	
42	Has the district selected an indicator for the monitoring of immunization safety?		
**Evidence of using data for action**			
43	Is there an analysis of HU data performed regularly with HU staff?	Analysis can be done within supervisory visits, meetings at district level, etc.<br>Explore the quality of analysis as well as the exhaustiveness of the HUs said to be analysed: none of them should be left out.	
44	Do you send regular monthly written feedback to the HUs?		

45	Are areas of low access identified and evidence of action taken to deal with it?	Discuss the importance and reasons for low access. How do the three strategies (fixed site, outreach and mobile teams) relate to the issue of access in the district?	
46	Have reasons for any high drop-out been identified, and are there plans/actions to deal with it?	Are there managerial practices that could be changed to reduce the drop-out rate?	
47	Is there monitoring of HU vaccine stock-outs? (A stock-out is an interruption in vaccine supply [for any vaccine].)	The manager should be able to say (based on written information) whether any HU has encountered a vaccine stock-out. If no vaccine stock-out is reported, ensure that the monitoring is possible and is being implemented.<br><br>Staff should be monitoring the level of reserve stocks and taking action if stock goes below a specified reserve level.	
48	Are there problems with completeness and timeliness of reports?	Are the late or incomplete reports usually from the same HUs. What was done to follow them up? What other actions were taken to encourage/induce timely reporting.	
49	Are the recommendations made for the last three supervisory visits followed up in subsequent visits?		
50	Has the monitoring of the selected immunization safety indicator been adequate during the last 12 months?		
51	Are surveillance and coverage data compared to look for inconsistencies and then followed up to understand why?		
