# Supplementary material for: Accuracy and quality of immunization data in Iran: findings from data quality self-assessment survey in 2017
Source: BMC Health Serv Res. 2019 Jun 11;19:371. doi: 10.1186/s12913-019-4188-9 (PMC6560874; doi:10.1186/s12913-019-4188-9)
Supplement: Supplementary file 5 — Timeliness and completeness of Pentavalent 3 and MMR1vaccines reporting (PDF 12 kb) [file 12913_2019_4188_MOESM5_ESM.pdf]

Timeliness and completeness of Pentavalent 3 and MMR1vaccines reporting

Year	Month	MMR1				Pentvalent 3			
		Timeliness		Completeness		Timeliness		Completeness	
		No	Yes	No	Yes	No	Yes	No	Yes
2015	January								
	February								
	March								
2016	April								
	May								
	June								
Sum									
